# Phosphaturic mesenchymal tumor with *de novo* liver metastases: a case report and literature review

**DOI:** 10.1177/17588359241232092

**Published:** 2024-03-06

**Authors:** Sami Dwabe, Warren Chow

**Affiliations:** Division of Hematology/Oncology, University of California Irvine, 101 The City Dr., Orange, CA 92868, USA; Division of Hematology/Oncology, University of California Irvine, Orange, CA, USA

**Keywords:** FGF23, FN1-FGF1 fusion protein, osteomalacia, paraneoplastic syndrome, phosphaturic mesenchymal tumor, tumor induced

## Abstract

Phosphaturic mesenchymal tumors (PMTs) are rare tumors that can cause tumor-induced osteomalacia (TIO) through overproduction of FGF23, a peptide hormone that causes renal phosphate wasting and reduced osteoblastic activity. The diagnosis of PMTs can be difficult to make as the presenting symptoms are non-specific. Although PMT is a rare entity, most cases are benign in nature, not requiring further intervention after surgery, as resection is typically curative. Here, we present a unique case of malignant PMT with *de novo* liver metastasis in a female patient who presented with TIO and underwent surgical resection of her primary lesion with subsequent regression of her liver metastasis. Moreover, we analyze a review of literature and discuss the importance of a timely diagnosis of this rare phenomenon. It is encouraged that providers strongly consider a diagnosis of PMT in patients with otherwise unexplained bone pain, fatigue, weakness, especially if accompanied with hypophosphatemia. Further studies are also warranted to identify prognostic factors that predict a PMT’s malignant potential as they may help identify possible therapeutic targets.

## Plain language summary

Phosphaturic mesenchymal tumor with spread to the liver: A case report and literature review

Phosphaturic mesenchymal tumors (PMTs) are rare tumors that can cause bone loss through the excess production of a signal named FGF23. This hormone causes the kidneys to lose phosphate and reduces the body’s ability to build new bone. PMTs are incredibly rare and difficult to diagnose especially since the presenting symptoms can be seen in several different diseases. Although PMT is rare, most cases are benign, only requiring surgery. We are presenting a unique case of a patient who presented with PMT in her right thigh and had evidence that the disease spread to her liver. Our patient underwent surgical resection of her thigh lesion and subsequently had improvement of her liver lesion. In our study, we analyze a review of literature and discuss the importance of a timely diagnosis of this rare phenomenon. It is encouraged that providers strongly consider a diagnosis of PMT in patients with otherwise unexplained bone pain, fatigue, weakness, especially if accompanied by low phosphate levels. Further studies are also warranted to identify prognostic factors that predict a PMT’s malignant potential as they may help identify possible therapeutic targets.

## Introduction

Tumor-induced osteomalacia (TIO) is a paraneoplastic syndrome characterized by widespread osteomalacia, often presenting with weakness, myalgias, bone pain, and frequent fractures. This phenomenon may be associated with phosphaturic mesenchymal tumor (PMT), a rare tumor that cause renal phosphate wasting through production of a peptide hormone known as fibroblast growth factor 23 (FGF23).^1^ FGF23 normally functions by regulating proximal tubule resorption of phosphate and inhibits the activity of 1-α-hydroxylase, which affects renal conversion of 25-hydroxyvitamin D to 1,25-dihydroxyvitamin D.^2,3^ FGF23 overexpression by PMTs, therefore, leads to excess urinary wasting of phosphate (through its effect on the proximal tubule), depletion of calcium and phosphate from the bones, and reduced osteoblastic activity (through its effects on vitamin D3 production).

Diagnosis of PMTs can often be difficult to make and are often delayed given the non-specific nature of the presenting symptoms.^4^ Moreover, even if there is a clinical suspicion of PMT, there are frequently challenges in identifying the tumor itself. Once a PMT is properly identified, treatment entails surgical resection, as it is usually curative.^4,5^ Although PMT is a rare entity, most cases are benign in nature, not requiring further intervention after resection.^4,5^ Here, we present a unique case of malignant PMT with *de novo* liver metastasis in a female patient who presented with TIO.

The reporting of this patient’s clinical case conforms to the CARE case report guideline and is provided as a supplementary material^6^ (Supplemental Material 1).

## Case

A 30-year-old female presented with chief complaint of muscle weakness. At the time, she was also being evaluated by endocrinology for irregular menses, elevated testosterone levels, and concern for Cushing syndrome. During her evaluation, she was discovered to have been inadvertently self-administering exogenous glucocorticoids for joint pain. She had been injecting medications she obtained from Mexico which contained vitamin-B complex, NSAIDs, and dexamethasone.

She had undergone a muscle biopsy that showed type 2 muscular atrophy which is seen in patients with long-term glucocorticoid use. Predictably, she had suppressed adrenocorticotropic hormone (ACTH) and cortisol levels that recovered after discontinuing the medication, although her presenting symptom of muscle weakness did not improve.

Eventually, the patient was found to have an elevated alkaline phosphatase of 159 U/L (normal: 44–147 U/L) and low phosphorus level of 1.5 mg/dL (normal: 2.5–4.5 mg/dL). She had a normal intact parathyroid hormone (PTH) level, normal thyroid function, and a low vitamin-D level that corrected with vitamin-D replacement. The patient was placed on Neutra Phos tablets, which improved her phosphorus level to 2.2 mg/dL, but had quickly regressed to 1.6 mg/dL after she self-discontinued the medication. A 24-h urinary phosphorus indicated that the patient was losing 1000 mg of phosphorus per day, despite her already significantly decreased serum phosphorus levels.

She underwent a nuclear medicine octreotide scan which showed no definite suspicious focal radiotracer activity to suggest a somatostatin positive tumor. A CT scan of her abdomen and pelvis was then done which showed an indeterminate 4.4 cm × 5.1 cm × 6.1 cm rounded mass in the right hepatic lobe initially concerning for hepatic adenoma (Supplemental Image 1). A PET/CT then identified a small suspicious FDG-avid lesion in the soft tissues of the posterior right thigh (Supplemental Image 2). The patient subsequently underwent an MRI with gadolinium contrast which redemonstrated the presence of the right posterior thigh and hepatic mass (Supplemental Image 3).

An image-guided biopsy of the mass lesion in the posterior right thigh then confirmed a diagnosis of PMT. The Ki-67 index of the biopsied mass was measured at only 5% indicating a low-grade tumor. An image-guided biopsy of her liver mass confirmed involvement of her liver with PMT. The biopsied liver mass was described by pathology as having a low mitotic rate, which was consistent with a metastasis from her posterior thigh mass. Eventually, the right posterior thigh mass was excised by orthopedic oncology with pathology confirming PMT with negative resection margins.

Given the tumor’s low Ki-67 index and low mitotic rate, chemotherapy was deferred (as it is typically reserved for higher grade tumors). Next-generation sequencing was ordered which identified an FN1–FGFR1 fusion gene. Microsatellite instability (MSI) was stable. Loss of heterozygosity and tumor mutation burden were low.

The patient’s phosphorus level improved from 1.6–2.2 mg/dL shortly after the surgery. An MRI abdomen 1 month after the resection of the mass showed a stability in the size of her liver metastases (Supplemental Image 4). She was then started on monthly burosumab-twza injections, an antibody which functions by blocking FGF23 activity, thus inhibiting its excess effect on phosphate metabolism.^7^ A DEXA scan was done which was consistent with low mineral bone density, with Z scores of: lumbar spine −1.4, left hip −1.6, and femoral neck −2.2. Metastasectomy of the liver lesion was discussed but ultimately deferred at this time given the patient’s overall improvement in symptoms.

Repeat imaging obtained 5 months after the surgery demonstrated an overall spontaneous decrease in the size of her liver metastases (Supplemental Image 5). Her phosphorus level at the time improved to 2.7 mg/dL with an alkaline phosphatase level of 143 U/L. A 24-h urinary phosphorus indicated that the patient is now losing only 500 mg of phosphorus per day, a 50% decrease from her baseline prior to intervention (Table 1).

Currently, the patient is doing well on observation with continued monthly burosumba-twza injections. She is being frequently monitored for any evidence of disease progression, which she has not demonstrated thus far following the resection of her primary tumor.

## Discussion

PMT is an extremely rare entity that typically presents as a benign neoplasm. Furthermore, the presentation of PMT with a malignant phenotype is even more uncommon. The majority of patients who are diagnosed with PMT will present with TIO, characterized by renal phosphate excretion, hypophosphatemia, and osteomalacia. The mechanism by which this occurs is through inappropriate secretion of FGF23 by PMTs.^1^ This pattern of elevated FGF23 and hypophosphatemia, particularly in a young individual, is seen in a broad differential of diseases, including PMT, x-linked dominant hypophosphatemic rickets, autosomal recessive hypophosphatemic rickets, osteoglophonic dysplasia, and McCune Albright syndrome.^8^

Moreover, Fibronectin 1 (FN1) is a gene that is responsible for encoding a protein that also plays a major role in cell adhesion, growth, and differentiation.^9^ It is commonly associated with the development of various types of cancer including lung, breast, thyroid, and gastric.^10,11^ The FN1–FGFR1 fusion protein (as seen in this patient’s molecular testing) is a hallmark driver of PMT. It was discovered in 2015 by Jen-Chief Lee *et al*., after identifying its presence in 9 out of 15 PMTs they studied.^12^ The same group also identified the presence of another novel fusion protein, FN1–FGF1 in PMTs. This suggested that the FGF1/FGFR1 pathway is paramount in driving PMT tumorigenesis.^13^

FN1 is a transcriptionally active secreted protein that contains a self-assembly mechanism. It has been discussed that increased expression of the FN1–FGF1 fusion protein functions like FGF1 secreted at high levels, which would subsequently affect phosphorus metabolism.^2^ In the case of the FN1–FGFR1 fusion protein, previous studies have discussed that FN1–FGFR1 proteins could use the self-assembly and fibronectin-binding domains of FN1 to dimerize and achieve a ligand-independent activation of the FGFR1 domain.^13^ Examples of both the FN1–FGF1 and FN1–FGFR1 fusion proteins are displayed in Supplemental Figure 1.

Either of the two mechanisms will converge with the activation of the FGFR1 signaling pathway and increased activity of FGF23 (either by excess ligand production from the FN1–FGF1 fusion protein or the ligand independent activation of the FN1–FGFR1 fusion protein). Currently, there are no agents that target this specific pathway.

In most cases, since PMT is benign in nature, excision of the tumor can resolve the presenting clinical symptoms without any recurrence of disease.^4^ There are rarely case reports, which describe a *de novo* malignant presentation of PMT.^14^ Of the few reported malignant PMTs, most have been described as a recurrence of an already resected PMT.^15^ Currently, there are no definite prognostic factors that predict a PMT’s malignant potential, though rapid growth and change in size have been discussed as possible measures for early malignant transformation, whereas a smaller size of the tumor tends to predict a benign clinical course.^15,16^ Our patient’s presentation is unique; in that, her PMT presented with associated *de novo*, biopsy-proven, liver metastases. Interestingly, it appears that the resection of her primary tumor may have potentially contributed to a spontaneous regression in her liver metastases, just 4 months after her initial surgery.

At this time, the mechanism by which her liver metastases had decreased in size is unclear. We speculate that this may be a response to the burosumab-twza injections (which targets the FGF23 pathway), or an immunogenic response sparked by the resection of her primary tumor. It is also possible that given the indolent nature of PMT, that this may be a normal fluctuation of her remaining tumor burden. As discussed in the literature review by Montenari *et al.*, patients with metastatic PMTs are understandably unlikely to be cured by local resection and may require continued medications or multiple surgeries.^17^ As previously mentioned, subsequent surgery was discussed for our patient. However, in her case, given the morbidity of a partial hepatectomy, the fact that her liver metastasis had been decreasing in size, and that her symptoms of hypophosphatemia had been improving, additional surgical intervention was deferred.

## Conclusion

PMT is an exceedingly rare entity that is incredibly difficult to diagnose, leading to challenges in treating the disease. The lack of specific presenting signs and symptoms, the lack of clinical suspicion, the indolent nature of the disease, and the challenge of identifying a primary tumor often leads to a significant delay in the diagnosis of PMT. If undiagnosed, patients can often present with profound osteomalacia leading to fragility skeletal fractures. This makes a timely diagnosis paramount. It is encouraged that providers strongly consider a diagnosis of PMT in patients with otherwise unexplained bone pain, fatigue, weakness, especially if accompanied with hypophosphatemia. We believe that further studies are also warranted to identify prognostic factors that predict a PMT’s malignant potential as it may help identify possible therapeutic targets.

## Supplemental Material

sj-docx-1-tam-10.1177_17588359241232092 – Supplemental material for Phosphaturic mesenchymal tumor with de novo liver metastases: a case report and literature review Supplemental material, sj-docx-1-tam-10.1177_17588359241232092 for Phosphaturic mesenchymal tumor with de novo liver metastases: a case report and literature review by Sami Dwabe and Warren Chow in Therapeutic Advances in Medical Oncology

## Declarations

**Ethics approval and consent to participate:** Exempt IRB approval (UCI IRB # 3435) was obtained prior to the writing of this case report and literature review. Patient provided verbal consent to have case report written on her clinical course.

**Consent for publication:** Patient provided verbal consent to have case report written on her clinical course. No identifiable information was included in the manuscript.

**Availability of data and material:** Not applicable.
