# Supplementary material for: Phosphaturic mesenchymal tumor with de novo liver metastases: a case report and literature review
Source: Ther Adv Med Oncol. 2024 Mar 6;16:17588359241232092. doi: 10.1177/17588359241232092 (PMC10919134; doi:10.1177/17588359241232092)
Supplement: sj-docx-1-tam-10.1177_17588359241232092 – Supplemental material for Phosphaturic mesenchymal tumor with de novo liver metastases: a case report and literature review [file sj-docx-1-tam-10.1177_17588359241232092.docx]

Supplementary Figures

Figure 1: A. Diagram of the FN-FGF1 fusion protein. As both components are secreted proteins, it is expected that increased expression of the FN1-FGF1 fusion protein functions, similar to native FGF1 secreted at excess levels. B. Diagram of the FN-FGFR1 fusion protein with preserved transmembrane and kinase domain. The FN domain functions to induce a ligand-independent activation of the FGFR1 domain.

| Lab | Pre-operative | Post-operative (4 months) |
| --- | --- | --- |
| Phosphorus (serum) | 1.6 mg/dL | 2.7 mg/dL |
| Phosphorus (24 hr urine) | 1000 mg/day | 500 mg/day |
| Alkaline Phosphatase | 159 U/L | 143 U/L |

Table 1: Significant lab values prior to and after surgical resection of primary thigh mass

Radiology images


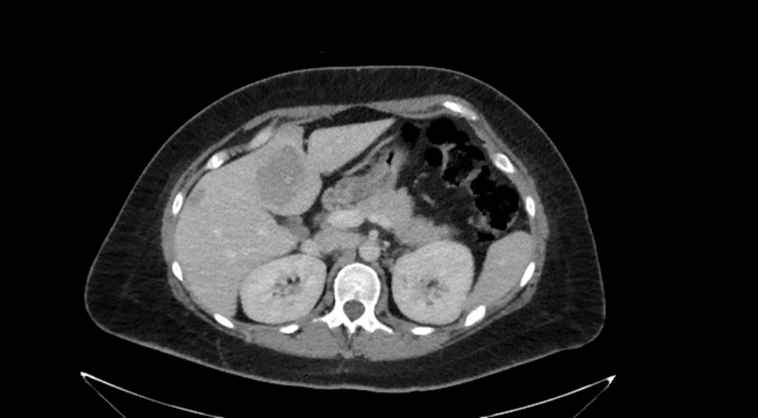

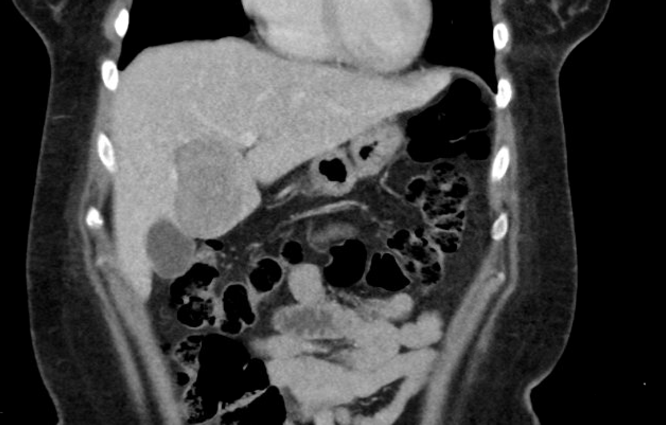


Image 1: CT Abdomen and Pelvis showing an indeterminate rounded mass in the right hepatic lobe concerning for hepatic adenoma


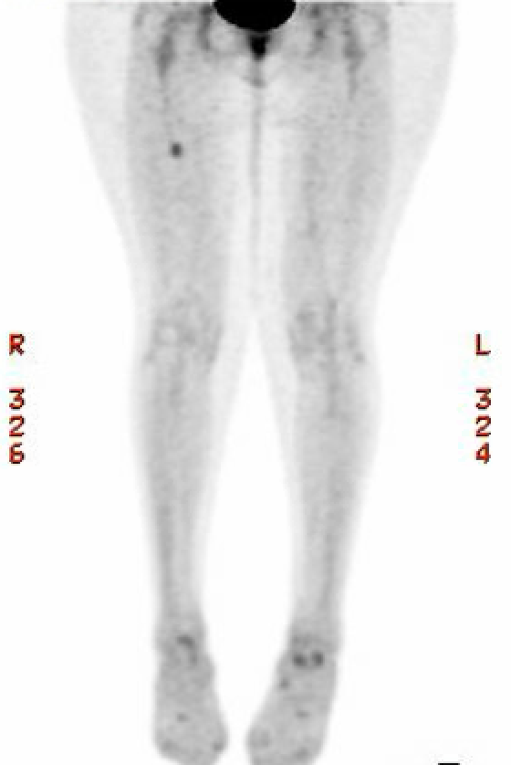


Image 2: PET/CT which identified a small suspicious FDG-avid lesion in the soft tissues of the posterior right thigh


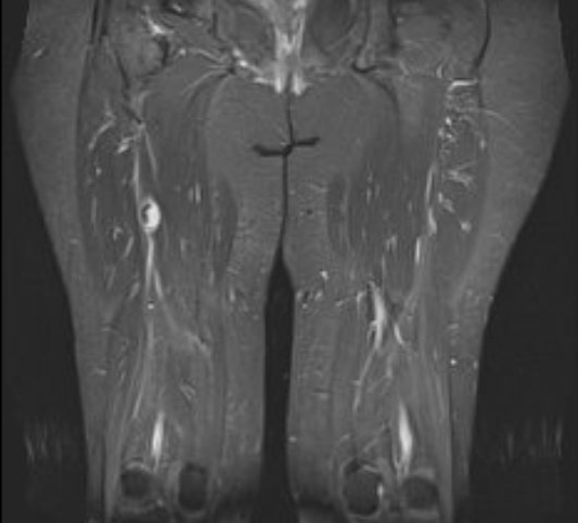

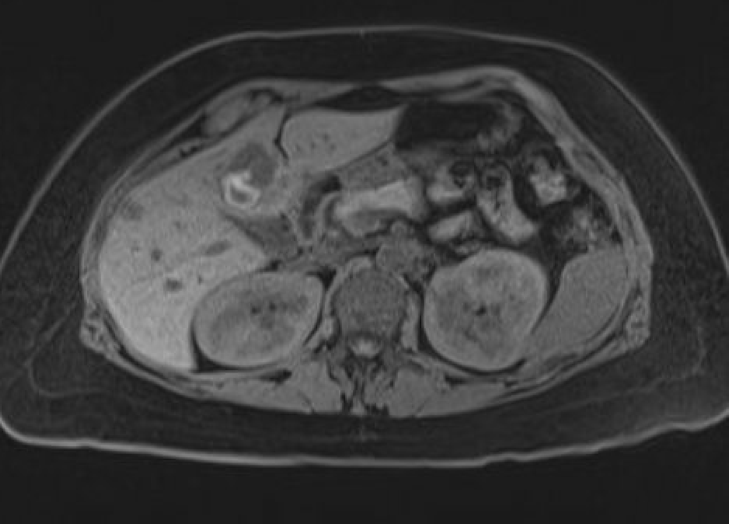


Image 3: MRI with gadolinium contrast which confirmed the presence of the right posterior thigh and hepatic mass


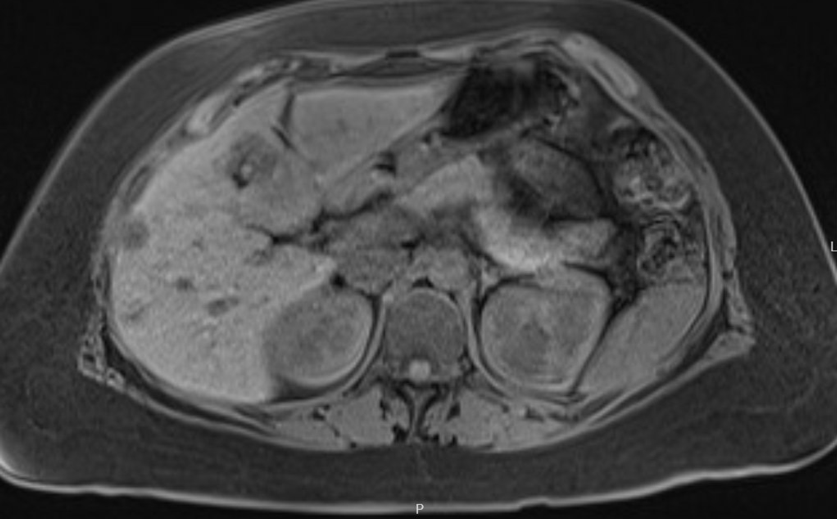


Image 4: Liver MRI one month following resection of right thigh mass


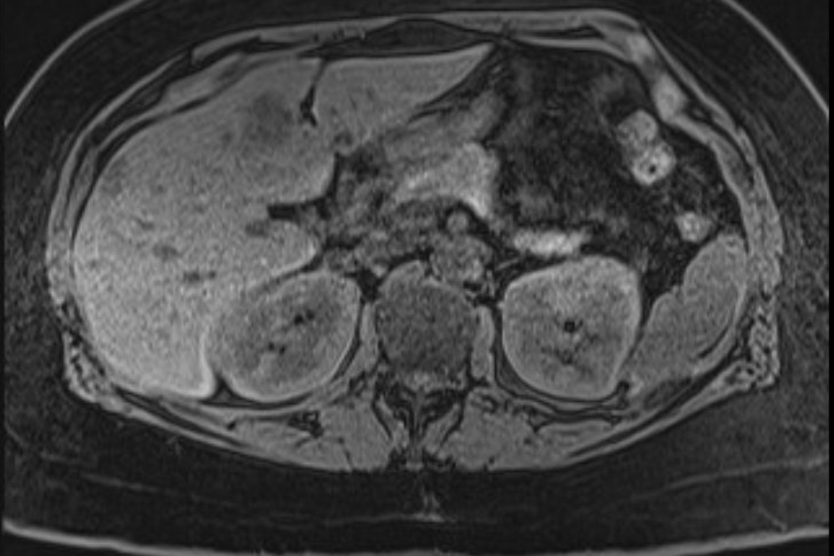


Image 5: Liver MRI five months following resection of right thigh mass
